# Development and optimization of a simian immunodeficiency virus (SIV) droplet digital PCR (ddPCR) assay

**DOI:** 10.1371/journal.pone.0240447

**Published:** 2020-10-09

**Authors:** Samuel Long, Brian Berkemeier

**Affiliations:** AIDS and Cancer Virus Program, Frederick National Laboratory for Cancer Research, Frederick, Maryland, United States of America; Consejo Superior de Investigaciones Cientificas, SPAIN

## Abstract

Accurate and sensitive quantification of rebound competent HIV that persists despite combination antiretroviral treatment (cART), including in latently infected cells (i.e., viral reservoir), is critical for evaluating cure strategies for decreasing or eliminating this reservoir. Simian immunodeficiency virus (SIV)-infected Rhesus macaques are an important non-human primate (NHP) system for studying potential cure strategies as they model many key aspects of human HIV-infection including the persistence of a latent viral reservoir in resting memory CD4+ T cells in animals receiving prolonged cART. In this report, we describe the design and testing of a sensitive SIV droplet digital PCR (ddPCR) assay through exploring the combination and optimization of different probe systems (including single, double quencher probes and minor groove binder (MGB) probes) and reaction conditions to eliminate background signal(s), ensure distinct target signal cluster separation from non-target signals, and enable detection and quantification of low level authentic target signals. Similar reaction conditions and assay validation procedures can be explored for potential development of additional assays for other applications that require sensitive detection of low-level targets in a large background of nucleic acid input derived from cell or tissue sources.

## Introduction

Droplet digital PCR (ddPCR) is a nucleic acid detection method that provides absolute quantification of specific targets by partitioning a standard quantitative PCR reaction into tens of thousands to millions of individual droplets of nanoliter or picoliter size. The system is designed to operate such that each droplet contains a single target molecule or no target. Sample partitioning allows sensitive, specific detection of single template molecules. The partitioning mitigates the effects of target competition, making ddPCR amplification less susceptible to inhibition and greatly improves the discriminatory capacity of assays.

One of the main advantages of ddPCR platforms compared to qPCR platforms is the capability for absolute quantification without the need for a standard curve. This feature allows effective comparison among quantitative measurements and quality control of routine analyses. Depending on how many droplets each reaction is partitioned into, ddPCR platforms vary in their sensitivity and precision. For example, the Raindance ddPCR system used in the current study partitions each reaction into 10 million droplets (i.e. up to a 6-log dynamic range in detection). In general, a platform or method with a wide dynamic range will enable linear detection of small-fold changes and can reliably count rare signals in a high background. The ability to be more tolerant to inhibitor(s) is another major advantage of ddPCR, as use of a digital positive or negative assay result rather than number of cycles to achieve a given threshold of reaction product signal accumulation removes the reliance on PCR amplification efficiency to reduce error rates. Due to these advantages, ddPCR has seen increased utility in fields that involve nucleic acid detection and quantification, which include but are not limited to rare allele/mutation detection [1–5], detection of pathogens including viruses [6–8], gene expression, miRNA analysis and copy number variation (CNV) determination [9–11], as well as absolute quantification of nucleic acid reference reagents, standards and NGS libraries [12].

Use of animal models is critical for evaluating the efficacy and mechanisms of novel therapeutics aimed at potential functional HIV cure [13,14]. Simian immunodeficiency virus (SIV)-infected Rhesus macaques receiving suppressive cART constitute a valuable NHP model system for studying HIV-1 infection, pathogenesis and potential cure strategies [15–21]. We recently used an ultrasensitive Raindance SIV ddPCR assay to overcome reaction inhibition and increase detection sensitivity when a large amount of DNA input was used in viral quantitation [6], as is often required in detecting low level viral DNA in tissue and cell samples from individuals receiving prolonged cART. Here we describe the experimental validation and optimization of this ddPCR assay. To optimize assay performance, during the development process of this assay, the following probe systems were evaluated, in addition to single quencher probes:

1. TaqMan MGB probes. MGB probes contain minor groove binder (MGB) moieties at the 3’ end which enable the probes to form very stable duplexes with the corresponding DNA targets. TaqMan MGB probes are usually significantly shorter than traditional probes in hybridization-based assays, and this feature can provide flexibility to accommodate more targets, and facilitate identifying potential probe design(s) in regions(s) of high conservation for assays that need to recognize multiple variants such as in the case of HIV-1. A/T rich duplexes in MGB probes are stabilized more than G/C rich duplexes, thereby leveling probe Tm and enabling simplification of design. The nonfluorescent quencher (NFQ) incorporated in TaqMan MGB probes absorb signals from the fluorescent dye label at the 5’ end of the probe, and this combined with the short length of the probe results in lower background signal (than with non-MGB probes such as with a single BHQ (black hole quencher)), which translates into increased sensitivity and data precision.
2. Double quencher probes. These probes include a second, internal quencher in the probe sequence to shorten the distance between the 5’ dye and quencher and, combined with the 3’ quencher, provide greater overall dye quenching, reduced background and increased signal detection compared to single quencher probes. The reduction in background allows using more probes in a multiplex qPCR experiment by decreasing crosstalk between channels; additionally, more than one probe can be used to assay for the presence of a gene in one detector channel, an ability that finds special utility in detecting infectious disease and biothreat agents in the field [22]. On platforms which have a fixed amount of “headroom” (i.e. the amount of overall signal allowable before saturation), the background from single quencher probes often results in “railing” (a phenomenon where the signal plateaus as the instrument does not have sufficient dynamic range to accommodate the later amplification process), which can lead to signal bleed over into adjacent channels and complicate data interpretation if those channels are also being used. Double quencher probes can resolve fluorescence saturation and bleed-over issue due to their low background. Double quencher probes also make it more feasible to design longer probes due to their lower background fluorescence. While traditional probes are restricted in length by the proximal quenching ability of the chosen quencher modification, by including an internal quencher longer probes can be designed to achieve a higher Tm, for example, when targeting regions of low complexity such as in AT-rich transcripts, thereby providing greater assay design flexibility. The increased sensitivity of double-quenched probes can be crucial for detecting limited targets due to small sample size or low expression, and the performance of these probes has enabled applications that would otherwise have not been possible [23].

In this report, we tested the ability of the MGB and double quencher probe assays to improve assay performance on the Raindance ddPCR system over the single quencher probe assays, especially in the context of sensitive detection of low level SIV DNA from cell and tissue samples in virus-infected Rhesus macaques that were on suppressive cART.

## Results

### Test samples

Four types of samples of increasing genetic background complexities were used during the assay validation and optimization process described herein. (1) Purified SIV DNA template (a plasmid containing a 918-bp region of the *gag* coding region derived from the full-length SIVmac239 molecular clone) [24]. (2) A mixture of the SIV DNA template and CCR5 DNA template (a plasmid containing a cloned genomic fragment of the Rhesus macaque CCR5 gene promoter region) [25]. This was a reduced complexity template. (3) Tissue DNA samples that were subjected to SIV nested PCR, and in which both SIV (nested) and CCR5 (unnested) quantities were determined using respective real time PCR assays. The advantage of this sample type was that the SIV signal was preamplified, and its corresponding ddPCR signal clusters were easy to detect and compare among different test conditions due to the large signal counts. (4) Unamplified tissue/cell samples. These samples were identical in genetic background complexity to test samples that are usually encountered in NHP research.

### Performance of single quencher probe assays on Raindance ddPCR platform

**Fig 1:**
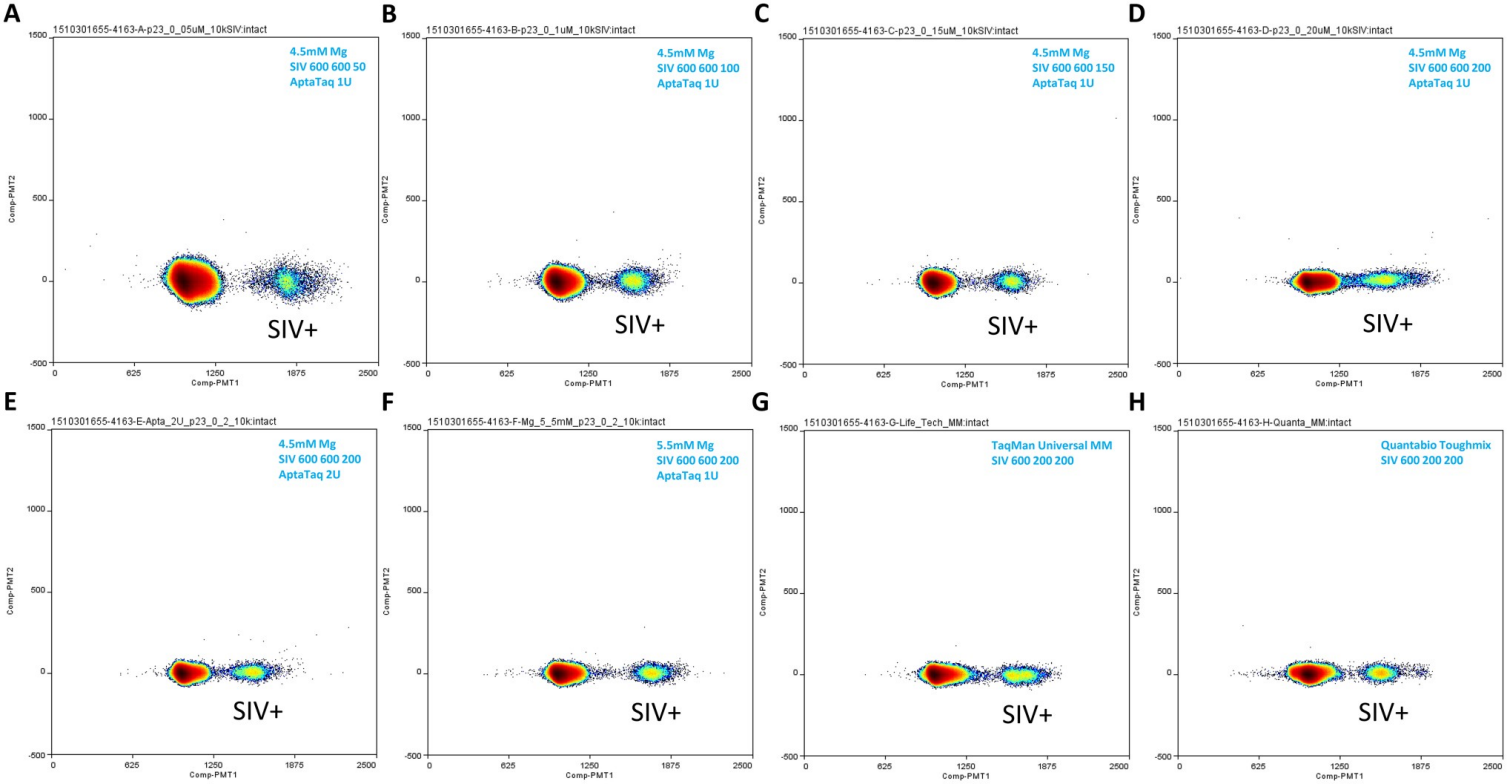
Performance of SIV single quencher probe assay on Raindance ddPCR. (A-D) Final SIV probe concentration varied from 50nM (A) to 200nM (D). (E) AptaTaq amount in this reaction was increased to 2U (compared to A-D and F, 1U AptaTaq in each reaction). (F) Final MgCl2 concentration was increased to 5.5mM (compared to A-E, 4.5mM MgCl2 in each reaction). (G) Single quencher probe SIV assay in TaqMan Universal mastermix. (H) Single quencher probe SIV assay in Quantabio Toughmix. For reactions performed in qPCR mastermix (A-F), MgCl2 concentration, primer, probe concentrations (in nM) and enzyme amount are indicated for each reaction in the corresponding plot’s upper right corner. Primer and probe concentrations were in the following order using 1A as an example: SIV (assay) 600 (nM, forward primer) 600 (nM, reverse primer) 50 (nM, probe). For reactions involving commercial mastermixes, only primer and probe concentrations are indicated. SIV DNA standard input in each reaction was 10000 copies. Additional reaction condition information (including template input and thermal cycling condition) is listed in Table 1.

**Fig 2:**
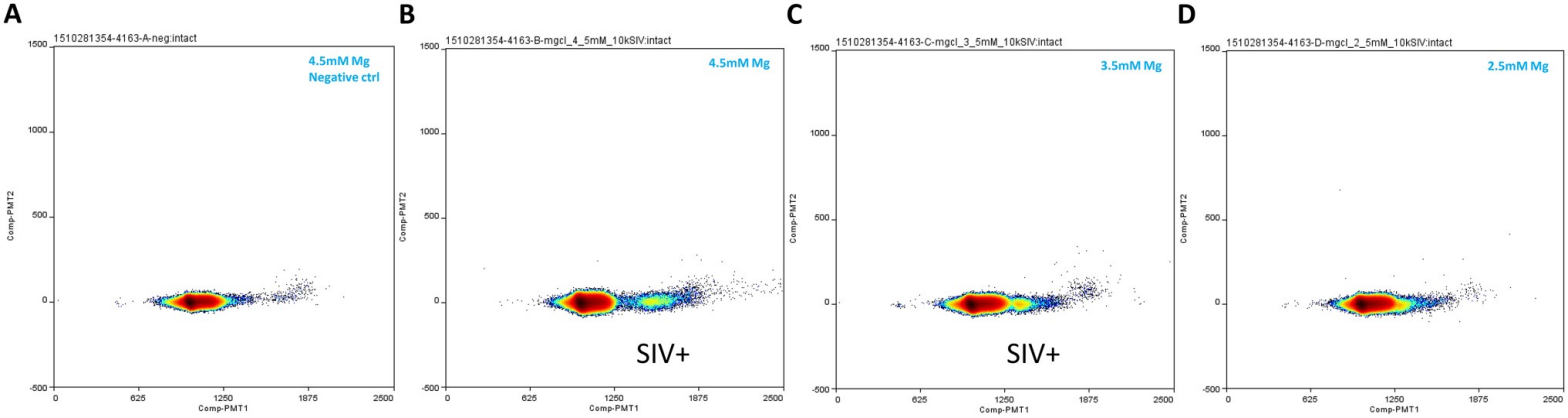
Single quencher probe assay Mg concentration test in the qPCR mastermix. (A) Negative control. (B-D) Final MgCl2 concentration varied from 4.5mM down to 2.5mM. MgCl2 concentration is indicated for each reaction in the corresponding plot’s upper right corner. AptaTaq amount in each reaction was 1U. SIV DNA standard input in each reaction in B-D was 10000 copies, and in the negative control reaction A, 0 copy. Additional reaction condition information (including thermal cycling condition) is listed in Table 1. SIV count in the ddPCR reactions was conducted for B only as in other reactions, SIV target region signals were due to background signals (A) and/or not separated well from the negative cluster (C and D).

**Fig 3:**
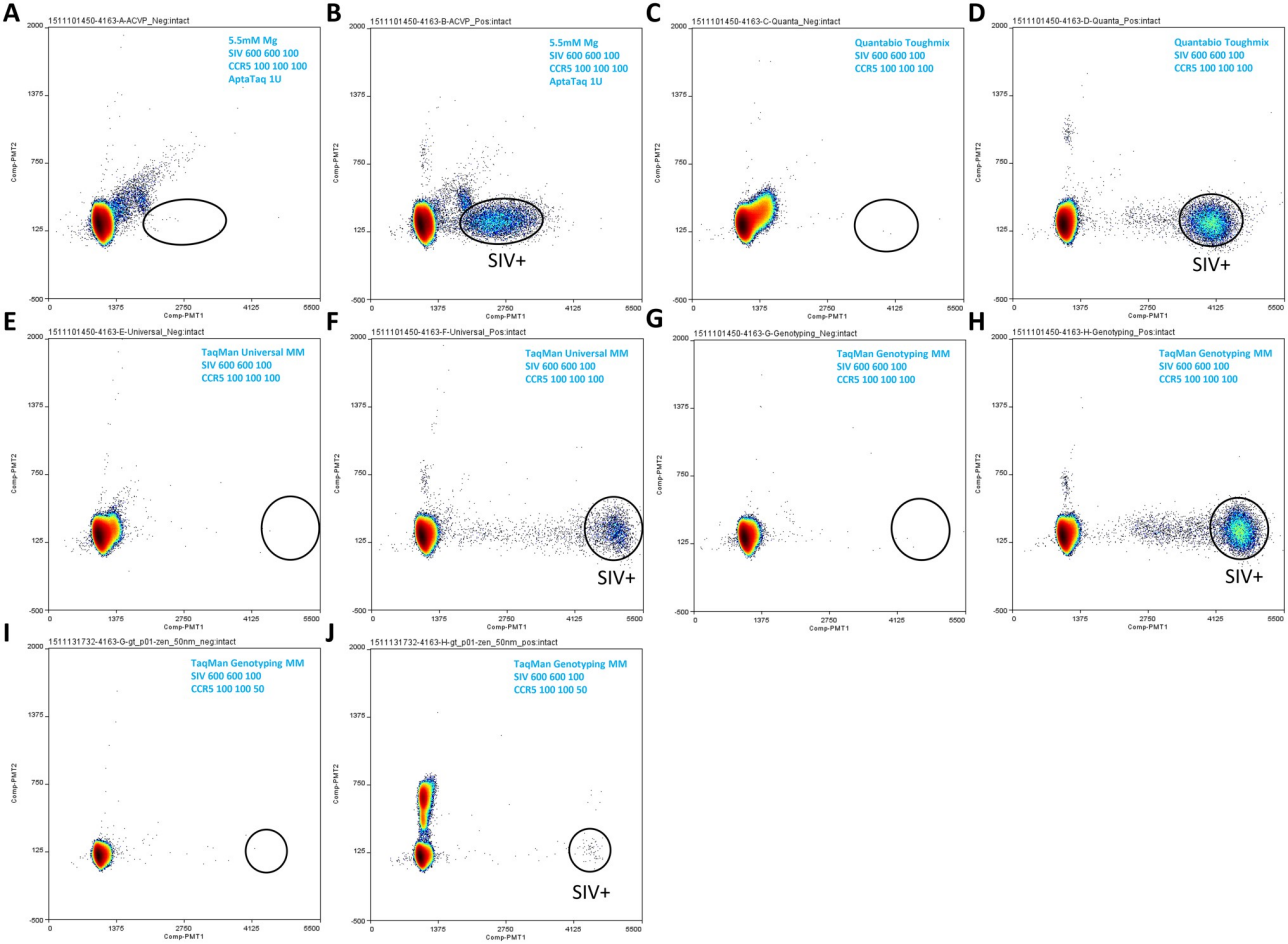
Double quencher probe assay ddPCR testing in different mastermixes. SIV and CCR5 double quencher probe assays were tested in duplex format in the following mastermix conditions: (A, B) qPCR mastermix; (C, D) Quantabio Toughmix; (E, F) TaqMan Universal mastermix; (G, H, I, J) TaqMan Genotyping mastermix. Mastermix condition and assay primer and probe concentrations for each reaction are indicated in the corresponding plot’s upper right corner. SIV DNA input (from SIV nested lymph node DNA from animal 311–04) in each reaction in B, D, F and H was 10000 copies, and in corresponding negative control reactions A, C, E, and G, 0 copy. SIV DNA standard input in J was 100 copies (in the background of 500000 copies of CCR5 DNA standard), and in corresponding negative control reaction I, 0 copy. Additional reaction condition information (including thermal cycling conditions) is listed in Table 1.

A sensitive qPCR assay was previously developed and used to detect and quantify low level SIV in cART-suppressed Rhesus macaques [26]. Our initial ddPCR assay development effort was aimed at adapting this assay to the Raindance platform with no or minimal changes introduced to the primer and probe sequences, or mastermix components, as direct transfer of qPCR assays onto ddPCR platforms was described [27,28]. As it was evident from this initial attempt that the SIV assay with its existing compositions (including mastermix) did not allow sufficient cluster separation [6], various changes were introduced to the existing mastermix composition to improve the performance of the single quencher probe assay on the Raindance platform. These included titrating MgCl2, and varying probe and enzyme concentrations (Figs 1 and 2 and S1 Fig). While the effect of AptaTaq enzyme quantity (compare Fig 1E (2U AptaTaq) to Fig 1D (1U AptaTaq)) on cluster separation was minor, decreasing probe concentration improves cluster separation appreciably (Fig 1A–1D: 50 to 200 nM probe concentration range; S1 Fig: 200 to 400 nM probe concentration range), although the lowest probe concentration tested, 50 nM, caused the SIV signal cluster to be more diffuse (Fig 1A) than higher probe concentration conditions. MgCl2 concentration by far had the largest influence on cluster separation (Fig 2). For example, we observed that compared to 4.5mM final MgCl2 concentration, there was much less distinct cluster separation when MgCl2 concentration was reduced to 3.5mM, and when MgCl2 concentration was further reduced to 2.5mM, the SIV cluster migrated further toward and merged with the negative cluster (Fig 2), likely due to inefficient PCR amplification. Two commercial mastermixes, TaqMan Universal mastermix and Quantabio Toughmix, allowed reasonable cluster separation (Fig 1G and 1H) that was comparable or slightly less than when 5.5 mM MgCl2 was included in the qPCR mastermix. 5.5 mM MgCl2 in the qPCR mastermix achieved the best cluster separation based on this mastermix condition (Fig 1F). However, a major issue associated with the single quencher probe assay in the qPCR mastermix (modified or unmodified) is significant background in the SIV target detection region (even when no SIV DNA template was present) (Figs 2A and 3A; S1A Fig). This limited the utility of using the single quencher probe assay in this mastermix for low level SIV viral detection (e.g. in viral reservoir/cure studies) on the ddPCR platform.

### Double quencher probe test for the SIV ddPCR assay

As direct transfer of the single quencher (blackhole quencher, BHQ) probe-based qPCR assay onto the Raindance ddPCR platform did not yield satisfactory performance, we considered switching to a double-quenched probe system. With double quencher probes, there is an additional quencher 9 base pairs from the 5’ dye in addition to the 3’terminal BHQ quencher. Dually quenched negative droplets are expected to provide increased signal-to-noise ratio allowing for better cluster separation. In evaluating assay and mastermix performance, we tested the double quencher probe assays in combination with several PCR mastermixes in addition to the existing qPCR mastermix. These include TaqMan universal mastermix, TaqMan genotyping mastermix, Quantabio Toughmix and AccuStart Genotyping Toughmix. These mastermixes were chosen due to their respective strengths such as robustness in generating assay signals, resistance to inhibition due to template impurities, and prior optimization for end point PCR etc. (see Material and methods section for details). In addition to cluster separation and background signal, parameters such as signal count (i.e. whether the positive droplet count agrees with the number of input target molecules) and signal cluster diffuseness under different mastermix conditions were also considered.

Upon conversion to the use of double quencher probes, the SIV and CCR5 assays (probes labeled with FAM and HEX, respectively) were duplexed (the CCR5 assay monitors cell equivalent total DNA input) and tested on the Raindance ddPCR platform in SIV-nested tissue samples (Fig 3A–3H) (SIV DNA viral load determined using the SIV qPCR assay), an SIV spike-in sample composed of 100 copies of SIV DNA template and half a million copies of CCR5 DNA template (Fig 3I and 3J), or non-nested tissue DNA sample (S2 Fig) (SIV DNA viral load determined using the SIV qPCR assay) (negative control reactions: Fig 3A, 3C, 3E, 3G and 3I and S2A Fig). Among the 5 mastermix conditions tested, Quantabio Toughmix and TaqMan Genotyping mastermix performed the best, as shown by tight SIV signal clusters that could be easily distinguished from the negative clusters, and the good agreement between SIV input amount and SIV counts from the signal clusters (Fig 3D, 3H and 3J; Table 1). The SIV DNA input and ddPCR count were also in good agreement when the reaction was performed in the qPCR master mix (Fig 3B; Table 1). However, two issues were observed with the qPCR mastermix in ddPCR: (1) The SIV signal cluster was not as well separated from the negative cluster (e.g. compare Fig 3B to 3D and 3F for cluster separation). (2) In the qPCR mastermix, the negative cluster contained two minor, “shoulder” clusters, one of which partly overlaps with the SIV signal cluster, and this complicates downstream data analysis and quantification. The main issue associated with the TaqMan Universal mastermix was that the SIV count from the signal cluster was only 28.4% of the input SIV amount (Fig 3F; Table 1), indicating that in about 70% of the droplets that contained SIV templates, the PCR reaction failed to produce sufficient fluorescent signals to be detected during the Raindance “Sense” step. In AccuStart Genotyping Toughmix (S2B Fig), in addition to the SIV and CCR5 signal clusters, there were at least 3 additional clusters of unidentified origin. The CCR5 cluster in this mastermix appeared to be composed of two clusters, which could be due to a SNP in the primer or probe binding region; however, we noticed that this second cluster was absent in other master mixes. In combination, these observations suggested non-specific recognition of additional targets in the Rhesus macaque genome or SIV genome by the assay(s) in the AccuStart Genotyping Toughmix. One common issue with the double quencher probes in all mastermixes tested was the presence of background signals in the target (i.e. SIV) detection region when no SIV DNA template was present (Fig 3 and S2 Fig, all “no template” panels). This would limit the utility of the double quencher assay(s) in low level SIV viral detection on the Raindance ddPCR platform.

**Table 1:** ddPCR reaction conditions and quantification results.

Figure	Mastermix	MgCl2 concentration (mM)	Primer and probe concentration (nM)	Enzyme	SIV input (copies)	SIV count (copies)	PCR thermal cycling condition
1A	qPCR	4.5	SIV 600 600 50	AptaTaq 1U	10000	8556	95°C 3min, 45x(95°C 30sec, 60°C 1min), 98°C 10min, 4°C hold
1B	qPCR	4.5	SIV 600 600 100	AptaTaq 1U	10000	7644	
1C	qPCR	4.5	SIV 600 600 150	AptaTaq 1U	10000	7884	
1D	qPCR	4.5	SIV 600 600 200	AptaTaq 1U	10000	8024	
1E	qPCR	4.5	SIV 600 600 200	AptaTaq 2U	10000	8029	
1F	qPCR	5.5	SIV 600 600 200	AptaTaq 1U	10000	7903	
1G	TaqMan Universal	Mg [con] in 1x MM	SIV 600 600 200	MM enzyme	10000	9946	
1H	Quantabio Toughmix	Mg [con] in 1x MM	SIV 600 600 200	MM enzyme	10000	11167	
2A	qPCR	4.5	SIV 600 600 300	AptaTaq 1U	0	N.D.	95°C 3min, 45x(95°C 30sec, 60°C 1min), 98°C 10min, 4°C hold
2B	qPCR	4.5	SIV 600 600 200	AptaTaq 1U	10000	6935	
2C	qPCR	3.5	SIV 600 600 200	AptaTaq 1U	10000	N.D.	
2D	qPCR	2.5	SIV 600 600 200	AptaTaq 1U	10000	N.D.	
3A	qPCR	5.5	SIV 600 600 100, CCR5 100 100 100	AptaTaq 1U	0	24	95°C 3min, 40x(95°C 30sec, 60°C 1min), 98°C 10min, 4°C hold
3B	qPCR	5.5	SIV 600 600 100, CCR5 100 100 100	AptaTaq 1U	10000	10400	
3C	Quantabio Toughmix	Mg [con] in 1xMM	SIV 600 600 100, CCR5 100 100 100	MM enzyme	0	2	
3D	Quantabio Toughmix	Mg [con] in 1xMM	SIV 600 600 100, CCR5 100 100 100	MM enzyme	10000	11354	
3E	TaqMan Universal MM	Mg [con] in 1xMM	SIV 600 600 100, CCR5 100 100 100	MM enzyme	0	2	
3F	TaqMan Universal MM	Mg [con] in 1xMM	SIV 600 600 100, CCR5 100 100 100	MM enzyme	10000	2842	
3G	TaqMan Genotyping MM	Mg [con] in 1xMM	SIV 600 600 100, CCR5 100 100 100	MM enzyme	0	4	
3H	TaqMan Genotyping MM	Mg [con] in 1xMM	SIV 600 600 100, CCR5 100 100 100	MM enzyme	10000	10615	
3I	TaqMan Genotyping MM	Mg [con] in 1xMM	SIV 600 600 100, CCR5 100 100 50	MM enzyme	0	2	95°C 7min, 40x(95°C 15sec, 60°C 1min), 98°C 10min, 4°C hold
3J	TaqMan Genotyping MM	Mg [con] in 1xMM	SIV 600 600 100, CCR5 100 100 50	MM enzyme	100	107	
4A	Quantabio Toughmix	Mg [con] in 1xMM	SIV 600 600 100, CCR5 100 100 50	MM enzyme	0	0	95°C 7min, 40x(95°C 15sec, 60°C 1min), 98°C 10min, 4°C hold
4B	Quantabio Toughmix	Mg [con] in 1xMM	SIV 600 600 100, CCR5 100 100 50	MM enzyme	100	70	
5A	TaqMan Genotyping MM	Mg [con] in 1xMM	SIV 600 600 200, CCR5 200 200 200	MM enzyme	0	0	95°C 10min, 40x(95°C 15sec, 60°C 1min), 98°C 10min, 4°C hold
5B	TaqMan Genotyping MM	Mg [con] in 1xMM	SIV 600 600 200, CCR5 200 200 200	MM enzyme	100	136	
5C	TaqMan Genotyping MM	Mg [con] in 1xMM	SIV 900 900 200, CCR5 200 200 200	MM enzyme	0	0	
5D	TaqMan Genotyping MM	Mg [con] in 1xMM	SIV 900 900 200, CCR5 200 200 200	MM enzyme	100	123	
5E	TaqMan Genotyping MM	Mg [con] in 1xMM	SIV 900 900 200, CCR5 900 900 200	MM enzyme	0	0	
5F	TaqMan Genotyping MM	Mg [con] in 1xMM	SIV 900 900 200, CCR5 900 900 200	MM enzyme	100	101	
5G	TaqMan Genotyping MM	Mg [con] in 1xMM	SIV 900 900 200, CCR5 400 400 200	MM enzyme	0	0	
5H	TaqMan Genotyping MM	Mg [con] in 1xMM	SIV 900 900 200, CCR5 400 400 200	MM enzyme	100	114	
5I	TaqMan Genotyping MM	Mg [con] in 1xMM	SIV 900 900 200, CCR5 900 900 200	MM enzyme	94	83	
5J	TaqMan Genotyping MM	Mg [con] in 1xMM	SIV 900 900 200, CCR5 900 900 200	MM enzyme	31	22	
5K	TaqMan Genotyping MM	Mg [con] in 1xMM	SIV 900 900 200, CCR5 900 900 200	MM enzyme	10	6	
5L	TaqMan Genotyping MM	Mg [con] in 1xMM	SIV 600 600 200, CCR5 400 400 200	MM enzyme	94	77	
5M	TaqMan Genotyping MM	Mg [con] in 1xMM	SIV 600 600 200, CCR5 400 400 200	MM enzyme	31	18	
5N	TaqMan Genotyping MM	Mg [con] in 1xMM	SIV 600 600 200, CCR5 400 400 200	MM enzyme	10	4	
5O	TaqMan Genotyping MM	Mg [con] in 1xMM	SIV 600 600 200, CCR5 200 200 100	MM enzyme	18	11	

### MGB probe test for the SIV ddPCR assay

**Fig 4:**
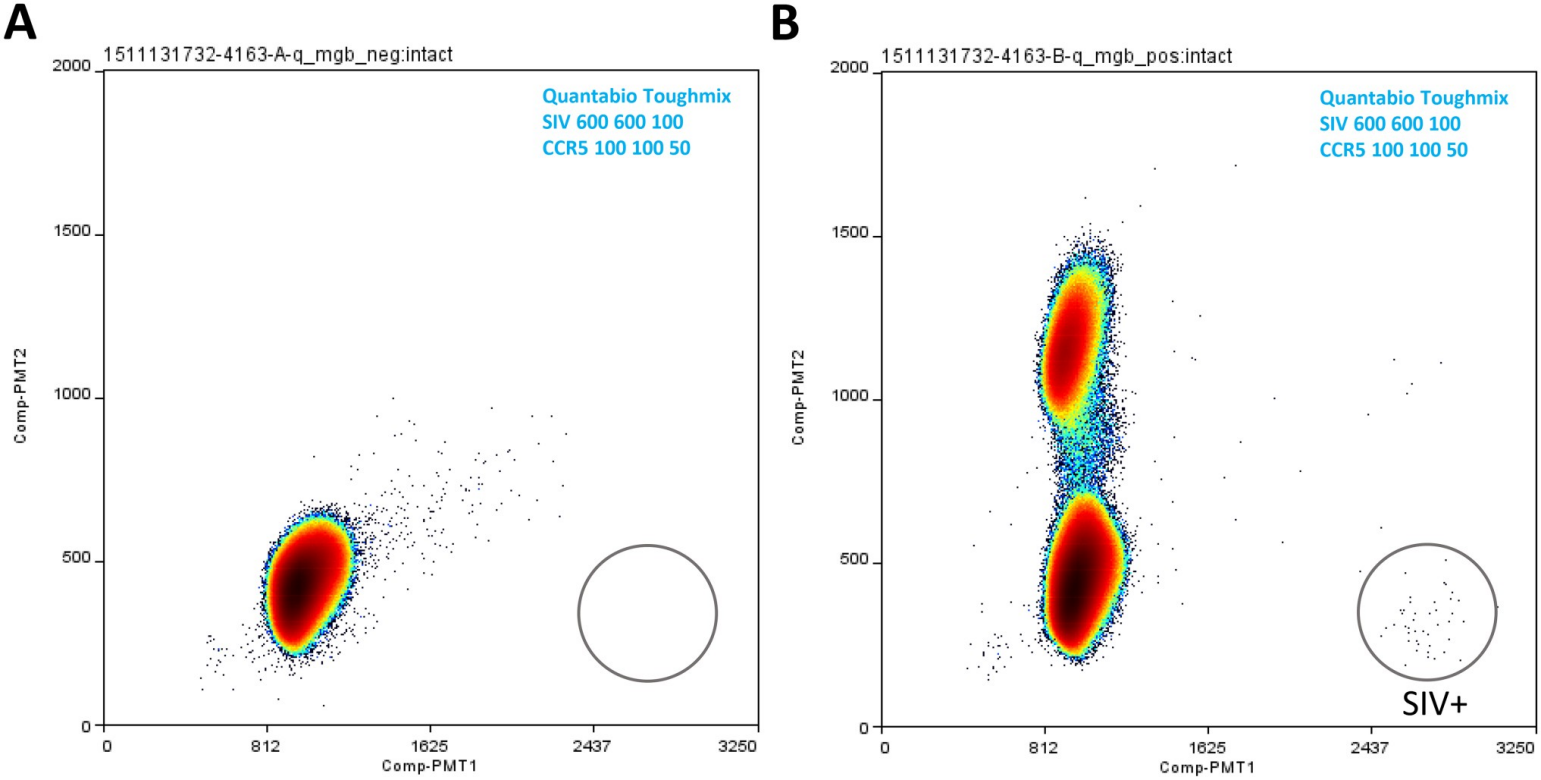
MGB probe assay ddPCR testing in Quanta Toughmix. SIV and CCR5 MGB probe assays were tested in duplex format in Quantabio Toughmix. Mastermix condition and assay primer and probe concentrations for each reaction are indicated in the corresponding plot’s upper right corner. SIV DNA standard input in B was 100 copies (in the background of 500000 copies of CCR5 DNA standard), and in corresponding negative control reaction A, 0 copy. Additional reaction condition information (including thermal cycling conditions) is listed in Table 1.

**Fig 5:**
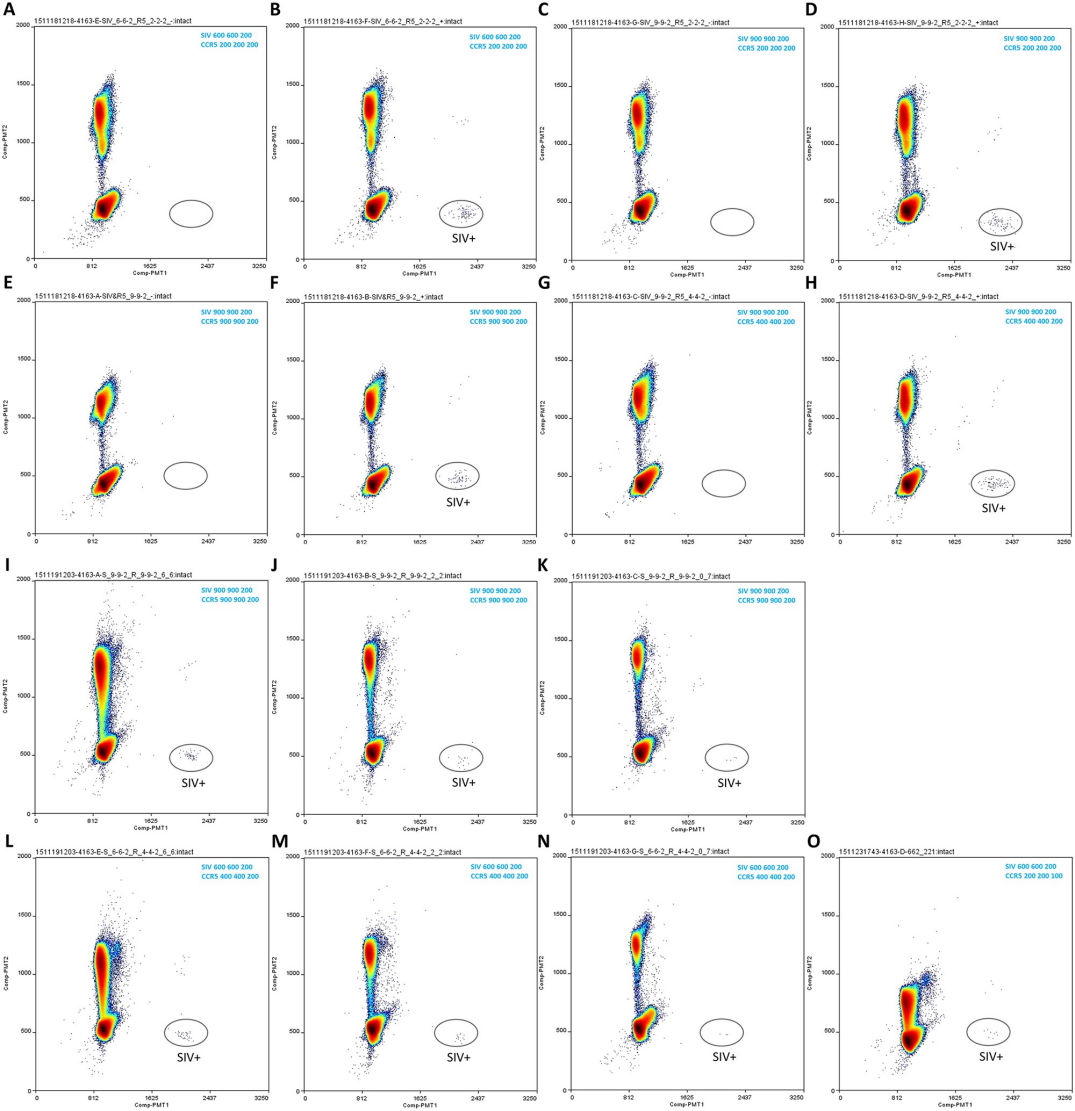
MGB probe assay ddPCR testing in TaqMan Genotyping mastermix. Different primer and probe concentration combinations were tested on spiked-in templates (A-H) and unnested tissue DNA (I-O). MGB assay primer and probe concentrations for each reaction are indicated in the corresponding plot’s upper right corner. SIV DNA standard input per reaction in B, D, F, H was 100 copies (in the background of 500000 copies of CCR5 DNA standard), and in corresponding negative control reactions A, C, E and G, 0 copy (in the background of 500000 copies of CCR5 DNA standard). SIV DNA input (from unnested ovary tissue DNA from animal 311–08) in I and L, J and M, K and N was 94, 31 and 10 copies, respectively. SIV DNA input (from unnested uterus tissue DNA from animal 313–08) in O was 18 copies. Additional reaction condition information (including thermal cycling condition) is listed in Table 1.

MGB probes (probes labeled with FAM (for SIV) and VIC (for CCR5) respectively) were then substituted in the SIV and CCR5 duplex assay for the double quencher probes and tested on Raindance ddPCR platform. The MGB probe assay performance was evaluated in three mastermix conditions, namely the Quantabio Toughmix and TaqMan Genotyping mastermix, which showed best performance for the double quencher probe assays, and AccuStart Genotyping Toughmix, developed for end-point PCR. The MGB probe assays performed reasonably well in the Quantabio Toughmix (Fig 4A and 4B), with good target cluster separation and no background signal in target area when target was not present. Significant under-quantification (input 100 copies, ddPCR count 70 copies) was however observed in this mastermix (also see “Discussion”). In AccuStart Genotyping Toughmix (S3A and S3B Fig), there were additional clusters of unidentified origin in addition to the SIV signal cluster and CCR5 signal cluster, again suggesting non-specific recognition of additional targets in the Rhesus macaque genome or SIV genome by the MGB assay(s) using this mastermix. TaqMan Genotyping mastermix proved to have the best performance among the three, yielding tight and distinct target clusters, no background signal with appropriate primer and probe concentrations, and signal count that is in agreement with input signal amount (Fig 5A and 5B; [6]).

Different primer concentration combinations of the SIV and CCR5 assays were tested (Fig 5: A-H, negative controls and SIV DNA spiked-in samples; I-O, unnested tissue DNA samples from infected animals). In general, these primer conditions performed similarly in cluster separation and quantitation (except O). It was observed that when higher concentrations (i.e. 900 nM each) of SIV assay primers were used, background signals in non-target areas increased. This was the case when spiked-in SIV DNA (Fig 5A–5H) or DNA from infected tissue samples (Fig 5I–5O) were used as template.

A “cluster-squeeze” phenomenon (i.e. two clusters were very close together) was observed with the MGB probe assay when unnested tissue DNA was used as the template and the CCR5 assay primer and probe concentrations were low (200 nM and 100 nM respectively) (Fig 5O), pointing toward potential reagent insufficiency (see “Discussion”). This issue could be resolved by increasing the primer and probe concentrations (compare Fig 5O and 5N).

## Discussion

The Raindance ddPCR system partitions a nucleic acid sample to up to 10 million picoliter-sized droplets, each containing a PCR reaction. Each droplet encapsulates a single target molecule to enable quick determination of the absolute counting of droplets containing positive fluorescent signal (specific target DNA). Following PCR amplification, every droplet is measured for fluorescence to generate a negative or positive signal, providing a digital result. Due to the high dynamic range of this platform, we previously adopted it for ultrasensitive detection of SIV in cART-treated Rhesus macaque, a non-human primate model of HIV-1 infected humans on anti-retroviral therapy. In DNA analysis, this platform was shown to be able to tolerate at least 35-fold more DNA input in each reaction compared to the BioRad ddPCR platform when the physical integrity of the droplets was examined [6]. Utilizing the optimized SIV ddPCR assay, each Raindance reaction also allowed 18-fold more DNA to be analyzed without observable inhibition [6], therefore enabling a significantly higher viral detection sensitivity. The current report documents the optimization process of this MGB probe-based SIV ddPCR assay involving investigating various probe systems and reaction conditions. This assay was also subjected to additional validation and testing [6]. The linear dynamic range of the assay in TaqMan Genotyping mastermix was at least up to 1 million copies (test upper limit) of viral nucleic acid per reaction. Applying the assay to detect ultralow level SIV viruses in tissue samples from cART-suppressed animals was also recently described [6].

Four main issues were observed with different probe-mastermix combinations. These include: (1) Low digital count compared to input template amount. This occurred under several conditions such as when double quencher probe assays were tested in TaqMan Universal mastermix and when MGB probe assays were tested in Quantabio Toughmix. This was likely due to inefficient PCR under the particular mastermix or cycling conditions. However, inaccuracy in qPCR quantification of the input template may have also contributed to the discrepancy between input and ddPCR count (see below). (2) Background signals in SIV target signal region when there was no SIV input. This problem was prominent when double quencher probes were combined with all mastermixes. This suggested potential hydrolysis of the probe which led to lack of complete probe quenching in the absence of target amplification. (3) Additional clusters of unidentified origin in addition to the specific, target signal clusters, pointing to the possibility of non-specific recognition of additional targets in the Rhesus macaque genome or SIV genome by the SIV and/or CCR5 assay under the specific mastermix condition(s). (4) Insufficient cluster separation between the positive cluster and negative cluster. This may again reflect inefficient PCR under the particular mastermix or cycling condition. The final condition that was chosen, namely MGB probe assays in the TaqMan Genotyping mastermix, was an assay combination that was void of these issues.

It is noteworthy that in this study, SIV template input quantity was based on quantitation of each respective template (be it SIV DNA standard template, SIV in nested tissue sample, or SIV in unnested tissue sample) with the SIV qPCR assay on a real time PCR platform, and such quantitation, being reliant on the use of external calibrators, can be subject to inaccuracies during the quantification and serial dilutions of the external calibrator molecules. For example, in Figs 3J, 4B, 5B, 5D, 5F and 5H, 100 copies of SIV DNA template were used as input in each reaction as determined by prior qPCR quantification. Based on ddPCR results in Fig 5B and 5D, the previous qPCR quantification of the SIV DNA template under-quantifies this template by about 26% (100 copies vs. 136 copies), assuming the condition in 5B allows detecting all target signals present. Similarly, inaccuracy in qPCR quantitation of SIV in unnested tissue samples such as used in Fig 5I to 5O may also have contributed to the discrepancy between inputs and ddPCR counts. Nevertheless, valid comparisons can still be made regarding the relative performance of the assays under various conditions (including mastermix conditions) by comparing the ddPCR signal counts obtained under these conditions.

A “cluster-squeeze” phenomenon (i.e. two clusters move closer to each other) was observed for the MGB probe assay when the CCR5 primer and probe concentrations were low, pointing toward a potential reagent (e.g. primers and probes) insufficiency. ddPCR on the Raindance platform segregates the reagents and templates of a 50 μL reaction into 10 million 5 picoliter-sized droplets. The advantage of this segregation is that the target templates can be enriched in certain droplets, greatly reducing the target template-containing droplets’ genetic background, therefore reducing competition and inhibition. A potential price of this segregation is that the reagents (primers, probe(s), enzyme and other components that are needed for the PCR reaction) are also evenly distributed into each of the droplet. Unlike in conventional real-time PCR, a reaction within each droplet in ddPCR is limited to the quantity of the reagents within the droplet. The relative sensitivity of the cluster separation (the distance of which reflects the positive reaction’s signal intensity) to primer and probe concentration is consistent with the possibility that the reagent amount for primers and probe(s) (e.g. at 600 nM and 200 nM, respectively) (and potentially other components in the reactions as well) are not in great excess as is the case in real time PCR reactions, in which the reaction components in the whole reaction volume are theoretically accessible to the positive reactions. For example, a 50μL PCR reaction containing 600 nM of each PCR primer will contain about 2 million copies of each primer in each droplet after dropletization. During the PCR step, assuming 100% PCR efficiency, the primers will be exhausted after 21 thermal cycles in a target-containing droplet. Similarly, in a PCR reaction that starts with 200 nM of each primer, the primers will be exhausted after ~20 thermal cycles in a target-containing droplet, and each positive droplet’s signal intensity on average will be about half of that of the previous reaction’s positive droplets. (A similar calculation can be made for the probe, which is another “consumed” reagent during the PCR reaction.) In applications where the CCR5 cluster signal is relied upon to quantify DNA input, “cluster-squeeze” can lead to inaccurate CCR5 quantification. Therefore, when feasible, DNA measurement (e.g. through NanoDrop) should be used as an independent parameter of template input. In addition, accurate counting of the two SIV-containing clusters (single occupancy cluster and dual occupancy cluster) is needed for duplex Poisson adjustment and calculation. We observed, however, that under the low CCR5 primer and probe concentration condition tested in Fig 5O, the two SIV-containing clusters were still reasonably well separated.

The MGB probe-based novel ddPCR assay forms the basis of sensitive detection and quantification of both SIV DNA and SIV RNA on the RainDance platform. As the field of HIV cure research continues to move forward, it has become increasingly apparent that the accurate and sensitive quantification of viral reservoirs is a key methodology that has yet to be refined. Without such methodologies, it is difficult to determine whether attempts at decreasing the size of the latent reservoir are successful; consequently, it is difficult to determine which interventions should be prioritized. As described in [6], the ddPCR assay described herein has the potential to mitigate many of the pitfalls that befall traditional attempts at low-level viral load quantification, namely, the large nucleic acid input that is required for low-copy viral load detection, and the presence of PCR inhibitors that are often present and difficult to remove. Of the advantages of the Raindance-based ddPCR assay, the most significant and well-characterized is the amount of genomic DNA that can be tolerated in a single reaction. In traditional ddPCR assays that use the BioRad platform, detection of viral DNA is significantly inhibited once the amount of input DNA exceeds 1.5μg (less than a quarter million mammalian cell equivalent). The optimized RainDance ddPCR assay presented here can tolerate up to 26.4μg of input DNA (4 million mammalian cell equivalent) per reaction without compromising droplet formation or showing reaction inhibition [6], thus making this assay ideally suited for the detection of rare events (such as SIV/HIV nucleic acids in the context of antiretroviral therapy in HIV reservoir/cure studies in which viral nucleic acid levels can be very low). Head-to-head comparisons of the RainDance ddPCR assay with standard qPCR for the quantification of SIV RNA showed that the presence of PCR inhibitors, such as heparin, do not interfere with viral nucleic acid detection in the RainDance assay. Importantly, we demonstrated the ability of the RainDance ddPCR assay to detect low level (e.g. single digit level) cell- and tissue-derived viral nucleic acids [6]. Therefore, this assay has many potential applications that will be of interest to the field especially to HIV reservoir/cure studies.

In conclusion, in this report we identified specific ddPCR assay conditions that form the basis for allowing ddPCR detection of SIV in Rhesus macaques and accurate measurement of viral nucleic acids especially from tissues and cells in infected animals undergoing cART treatment. Similar conditions can be explored on the Raindance ddPCR system to enable potential development and validation of additional assays for applications that require sensitive detection of low amount target(s) from a background of large nucleic acid input derived from cell or tissue sources.

## Materials and methods

### DNA extraction and qPCR quantification of SIV viral DNA

In vivo derived specimens were as described in [6]. DNA isolation and preamplified qPCR quantification of cell and tissue-derived SIV viral DNA followed procedures and conditions published previously [6,25,29,30]. Briefly, DNA was purified following TriReagent (Molecular Research Center) manufacturer’s recommended back-extraction method with modifications as described in [6]. qPCR quantification of SIV viral DNA followed a nested qPCR protocol [6, 24,25,29] and real time PCR quantification was performed using on ViiA 7 real time PCR system (ThermoFisher Scientific).

### ddPCR improvement based on single quencher probe assays and qPCR mastermix

For single quencher probe assay ddPCR testing, the primer and probe sequences were as follows: (1) SIV assay. SGagForward: GTCTGCGTCAT(dP)TGGTGCATTC; SGagReverse: CACTAG(dK)TGTCTCTGCACTAT(dP)TGTTTTG; SGagProbe: FAM- CTTC(dP)TCAGT(dK)TGTTTCACTTTCTCTTCTGCG-BHQ whereas dP and dK bases [31] denote non-standard bases (Glen Research, Sterling, VA) introduced to minimize the impact of potential sequence mismatches at positions of described heterogeneity in SIV ioslates (Los Alamos Sequence Database, http://hiv-web.lanl.gov/). (2) CCR5 assay. RCCR5Forward: CCAGAAGAGCTGCGACATCC; RCCR5Reverse: GTTAAGGCTTTTACTCATCTCAGAAGCTAAC; RCCR5Probe: VIC- TTCCCCTACAAGAAACTCTCCCCGGTAAGTA-BHQ. Single quencher probes were synthesized at Biosearch Technologies.

Each ddPCR reaction (25μL or 50μL) is composed of the following: MgCl2 (concentration varies), dNTPs (300 μM each), dUTP (600 μM), SGagForward (concentration varies), SGagReverse (concentration varies), SGagProbe (concentration varies); 1x PCR II buffer (ThermoFisher) with 0.2% Tween, AptaTaq (amount varies) or TaqGold (Perkin Elmer) polymerase, DNA template, 1xddPCR stabilizer (Raindance) and H2O. In SIV and CCR5 duplex ddPCR reactions, RCCR5Forward (concentration varies), RCCR5Reverse (concentration varies) and RCCR5Probe (concentration varies) were also included. Reagent concentration, enzyme amount, template input and thermal cycling condition for each test are listed in Table 1 & S1 Table.

### ddPCR optimization based on MGB and double quencher probe assays under various mastermix conditions

MGB and double quencher probe ddPCR assay performance was tested and optimized in several PCR mastermixes in addition to the qPCR mastermix. These include: (1) TaqMan Universal mastermix (ThermoFisher Scientific), (2) Quantabio Toughmix (Quantabio), (3) TaqMan genotyping mastermix (ThermoFisher Scientific) and (4) AccuStart genotyping Toughmix (Quantabio). TaqMan Universal PCR Master Mix Contains AmpliTaq Gold DNA Polymerase and buffer enhancement to increase signal yield and assay robustness in applications including those involving G/C-rich sequences. The Quantabio Toughmix was chosen because this mastermix performed well (albeit on qPCR platforms) in applications where PCR inhibition from template impurities may be a concern, such as crude extracts, clinical specimens, or environmental samples. TaqMan genotyping mastermix and AccuStart Genotyping Toughmix are mastermixes that were optimized for end point PCR.

For double quencher probe assay ddPCR testing, the primers and probes used were as follows: (1) SIV assay. SGagForward and SGagReverse were used as forward and reverse primers respectively. SGagProbeDQ sequence was FAM-CTTCYTCAG(Zen)TRTGTTTCACTTTCTCTTCTGCG-IABkFQ with Y and R being bases for C/T and A/G mixes respectively. (2) CCR5 assay. RCCR5Forward and RCCR5Reverse were used as forward and reverse primers respectively. RCCR5ProbeDQ sequence was HEX-TTCCCCTAC(Zen)AAGAAACTCTCCCCGGTAAGTA-3IABkFQ. Double quencher probes were synthesized at Integrated DNA Technologies, Inc.

For MGB probe assay ddPCR testing, the primers and probes used were as follows: (1) SIV assay. SGagForward and SGagReverse were used as forward and reverse primers respectively. SGagProbeMGB sequence was 5’-FAM- CTT CYT CAG TRT GTT TCA CTT T -MGB with Y and R being bases for C/T and A/G mixes respectively. (2) CCR5 assay. RCCR5Forward and RCCR5Reverse were used as forward and reverse primers respectively. RCCR5ProbeMGB sequence was VIC- TTC CCC TAC AAG AAA CT-MGB. Taqman MGB probes were synthesized at ThermoFisher Scientific.

For double quencher probe and MGB probe assay test in the 4 commercial mastermixes, each ddPCR reaction (50μL) was composed of the following: 1x mastermix (varies), SGagForward (concentration varies), SGagReverse (concentration varies), SGagProbeDQ or SGagProbeMGB (concentration varies), DNA template, 1xddPCR stabilizer (Raindance) and H2O. In SIV and CCR5 duplex ddPCR reactions, RCCR5Forward (concentration varies), RCCR5Reverse (concentration varies) and RCCR5ProbeDQ or RCCR5ProbeMGB (concentration varies) were also included. The mastermix, reagent concentration, template input and thermal cycling condition for each test are listed in Table 1. Double quencher probe test in the qPCR mastermix was performed as described in the “ddPCR improvement based on single quencher probe assays and qPCR mastermix” section above except that double quencher probes were used instead of single quencher probes. The reagent concentrations, enzyme amount, template input and thermal cycling condition for each reaction is listed in Table 1 and S1 Table.

### ddPCR

Droplet generation (i.e. dropletization), end point PCR, Raindance Sense instrument reading of droplet fluorescence signals, and data analysis were performed as described in [6].

### In vivo derived specimens

Specimens were graciously provided by Dr. Louis Picker (Oregon Health and Science University) and Dr. Paul Johnson (Emory University), from animals in protocols approved by the Institutional Animal Care and Use Committees at their respective institutions (Oregon National Primate Research Center’s Animal Care and Use Committee, and Emory University Institutional Animal Care and Use Committee, respectively). All experiments were performed in accordance with relevant guidelines and regulations.

## Supporting information

S1 Fig SIV single quencher probe assay probe and primer concentration test in qPCR mastermix. (DOCX)

S2 Fig Double quencher probe assay ddPCR testing in AccuStart Genotyping Toughmix. (DOCX)

S3 Fig MGB probe assay ddPCR testing in AccuStart Genotyping Toughmix. (DOCX)

S1 Table ddPCR reaction conditions and quantification results. (XLSX)
