# Supplementary material for: Development and optimization of a simian immunodeficiency virus (SIV) droplet digital PCR (ddPCR) assay
Source: PLoS One. 2020 Oct 9;15(10):e0240447. doi: 10.1371/journal.pone.0240447 (PMC7546489; doi:10.1371/journal.pone.0240447)
Supplement: S1 Fig — (DOCX) [file pone.0240447.s001.docx]

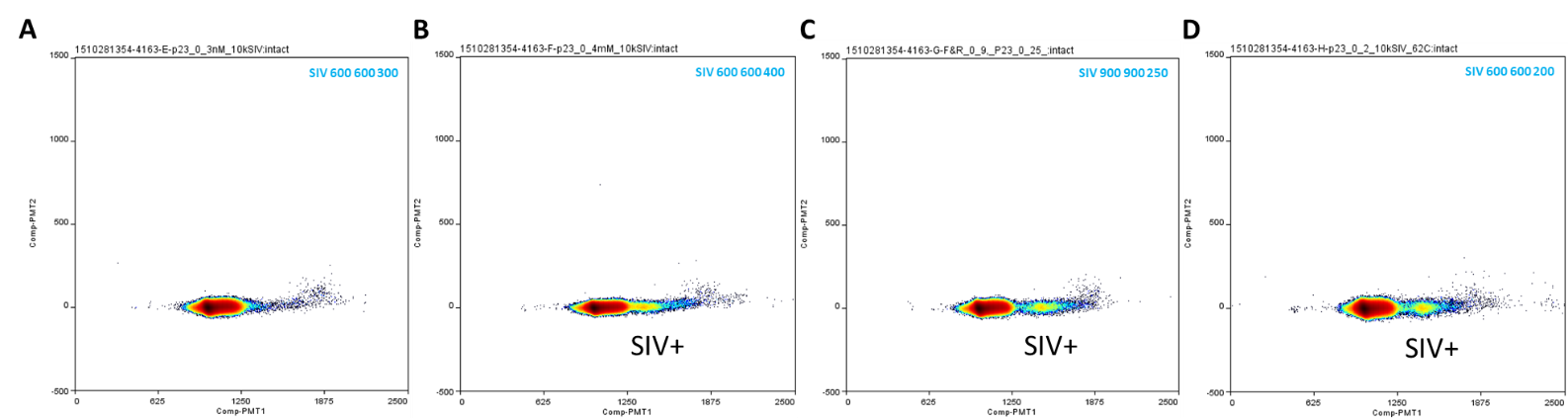


**Supplemental Figure 1. SIV single quencher probe assay probe and primer concentration test in qPCR mastermix.** Primer and probe concentrations are indicated for each reaction in the corresponding plot’s upper right corner. AptaTaq quantity in each reaction was 1U and the final MgCl2 concentration in each reaction was 4.5 mM. SIV DNA standard input in each reaction in B-D was 10000 copies, and in the negative control reaction A, 0 copy. Additional reaction condition information (including thermal cycling conditions) is listed in Supplemental Table 1. Note the background signal in SIV target signal region in (A) when there was no SIV DNA template included in the reaction. SIV count in the ddPCR reactions was conducted for (C) and (D) only as in other reactions, SIV target region signals were impinged upon by background signals (A) and/or not separated well from the negative cluster (B).
