# Supplementary material for: Development and optimization of a simian immunodeficiency virus (SIV) droplet digital PCR (ddPCR) assay
Source: PLoS One. 2020 Oct 9;15(10):e0240447. doi: 10.1371/journal.pone.0240447 (PMC7546489; doi:10.1371/journal.pone.0240447)
Supplement: S2 Fig — (DOCX) [file pone.0240447.s002.docx]

**
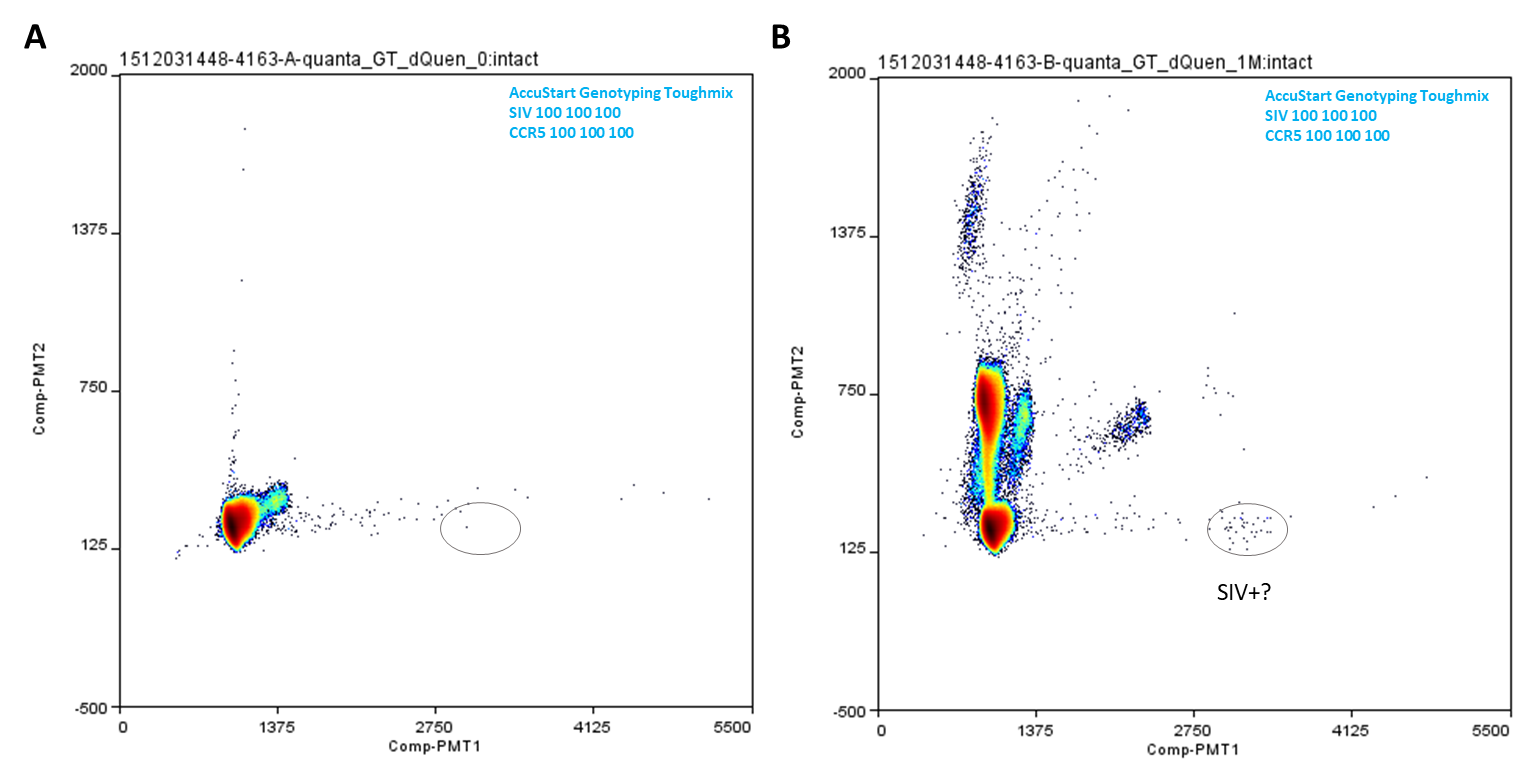
**

**Supplemental Figure 2. Double quencher probe assay ddPCR testing in AccuStart Genotyping Toughmix.** SIV and CCR5 double quencher probe assays were tested in duplex format in AccuStart Genotyping Toughmix. Mastermix condition and assay primer and probe concentrations for each reaction are indicated in the corresponding plot’s upper right corner. SIV DNA input (from 1 million cell equivalent of unnested ovary tissue DNA from animal 311-08) in B was 93 copies, and in corresponding negative control reaction A, 0 copy. Additional reaction condition information (including thermal cycling conditions) is listed in Supplemental Table 1. SIV count in the ddPCR reactions was not conducted due to the presence of multiple clusters.
